# Is chronic hepatitis B infection a protective factor for the progression of advanced pancreatic ductal adenocarcinoma? An analysis from a large multicenter cohort study

**DOI:** 10.18632/oncotarget.13000

**Published:** 2016-11-01

**Authors:** Qiwen Chen, Zhouyu Ning, Lei Wang, Haifeng Ying, Shu Dong, Chenyue Zhang, Xiaoheng Shen, Yuanbiao Guo, Hao Chen, Xiaoyan Zhu, Yehua Shen, Weidong Shi, Yongqiang Hua, Kun Wang, Junhua Lin, Litao Xu, Lianyu Chen, Lanyun Feng, Xiumei Zhang, Jing Xie, Bo Sun, Yaqin Sun, Wenchao Gu, Mei Kang, Zheng Tang, Zhujun Chen, Zhen Chen, Luming Liu, Jinming Yu, Zhaoshen Li, Zhiqiang Meng

**Affiliations:** ^1^ Department of Integrative Oncology and Department of Oncology, Fudan University, Shanghai Cancer Center, Shanghai Medical College, Shanghai, China; ^2^ Institute of Clinical Epidemiology, Key Laboratory of Public Health Safety, Ministry of Education, School of Public Health, Fudan University, Shanghai, China; ^3^ Digestive Endoscopy Center, Department of Gastroenterology, Shanghai Changhai Hospital, Second Military Medical University, Shanghai, China; ^4^ Department of Integrative Medicine of Shanghai Ruijin Hospital, Shanghai Jiao Tong University School of Medicine, Shanghai, China; ^5^ Department of Endoscopy and Department of Oncology, Fudan University, Shanghai Cancer Center, Shanghai Medical College, Shanghai, China; ^6^ Department of Radiology and Department of Oncology, Fudan University, Shanghai Cancer Center, Shanghai Medical College, Shanghai, China; ^7^ Department of Clinical Laboratory and Department of Oncology, Fudan University, Shanghai Cancer Center, Shanghai Medical College, Shanghai, China

**Keywords:** pancreatic adenocarcinoma, HBV

## Abstract

**Purpose:**

Whether the progression of advanced pancreatic ductal adenocarcinoma (PDAC) patients could be affected by HBV exposure remains to be determined. Therefore, we conducted this study to assess the effect of HBV infection on PDAC progression among a large cohort in China.

**Methods:**

A multicenter cohort study was conducted to explore whether liver metastasis and overall survival in locally advanced and metastatic PDAC could be affected by HBV infection. In this study, we collected 1,526 advanced PDAC patients at three participating hospitals - Shanghai Cancer Center, Changhai Hospital and Ruijin Hospital from 2004 to 2013. The association between HBV status and advanced PDAC progression was then examined.

**Results:**

In multivariable Logistic regression model, chronic hepatitis B(CHB) infection was inversely associated with synchronous liver metastasis compared to non HBV infection (OR 0.41, 95% CI 0.19-0.85) for stage IV patients. In a multivariable Cox model, CHB infection (HR=0.11, 95% CI 0.02-0.82) is considered as a protective factor of metachronous liver metastasis compared to Non HBV infection for stage III patients. For stage IV patients, CHB infection was inversely associated with overall survival compared to non HBV infection (HR 0.70, 95% CI 0.51-0.95). Inactive carrier(IC) and resolved HBV infection showed no significant association with survival compared to non HBV infection.

**Conclusion:**

This study indicated that CHB infection may serve as an independent factor which decrease synchronous or metachronous liver metastasis, and increase overall survival among advanced PDAC patients.

## INTRODUCTION

Pancreatic ductal adenocarcinoma (PDAC) is the fourth leading cause of cancer deaths in the United States and the seventh leading cause of cancer deaths in China [1, 2]. Due to the obscure symptoms at early stages and the aggressive progression of the disease, only around 15-20% of PDAC patients have a chance to receive radical surgery at diagnosis and most of the patients are detected the disease with unresectable advanced tumors [3]. Due to the limited options for patients with PDAC at locally advanced and metastatic stages, these patients with unresectable PDAC often have dismal prognosis. Mounting evidences have demonstrated that some clinical parameters may affect the survival of PDAC patients such as tumor stages, postoperative pathological findings and CA-199 level [4-6]. However, to date whether hepatitis B virus (HBV) infection could influence the prognosis in patients with advanced PDAC has not been well established and confirmed yet.

China is one of the countries boasting high endemicity of HBV infection [7]. Roughly 7.2% of the Chinese population are chronic HBsAg carriers and approximately 60% have been exposed to HBV infection [ 8]. Thus, there is a great likelihood that some PDAC patients are also HBsAg carriers or they once were subject to HBV exposure. HBV is a well-known oncogenic virus for hepatocellular carcinoma (HCC) [ 9], which may also cause some extrahepatic malignancies or diseases, such as non-Hodgkin lymphoma [10, 11], aplastic anemia [12] and nephritis [13]. The presence of HBV infection in extra-hepatic sites such as pancreas and other organs has been identified in multiple studies [14-16].

Recently, opinions abound on whether there is an association between HBV infection and risk of PDAC but with no consistent conclusion has been reached on this issue [17-19]. Some studies have demonstrated that HBV infection may decrease the risk of liver metastasis in colorectal cancer (CRC) [20, 21]. Some studies showed that CRC patients with HBV infection may survive longer than those without [22, 23]. Whether the prognosis of advanced PDAC could be affected by HBV exposure remains to be determined, the result of which would be of great significance for future clinical practice. Herein, we conducted this multicenter cohort study to explore whether liver metastasis and survival in locally advanced and metastatic PDAC could be affected by HBV infection.

## PATIENTS AND METHODS

### Study populations

From January 1, 2004, to December 31, 2013, we conducted a multicenter cohort of patients with unresectable PDAC, including stage III and IV cases, at three participating hospitals, Shanghai Cancer Center(SCC), Changhai Hospital(CHH) and Ruijin Hospital(RJH). All the patients included were pathologically proved PDAC. In this study, data from 1526 patients with serologic assay for HBV infection in the cohort were finally analyzed.

This study was carried on in accordance with the precepts of theHelsinki Declaration. Approvals were obtained from the Human Investigations Committee at all the participating hospitals. During the hospital stay, every included patient or their guardians signed the informed consent in view of prospective research of the clinical data.

### Exposure measurements

#### HBV status

 Upon the initial admission to the in-patient department, blood samples were obtained from every patient. Based on the positivity of five routine markers (HBsAg, HBsAb, HBeAg, HBeAb, HBcAb) and HBV-DNA levels, we categorized patients into four groups as following: patients with no infection, patients with chronic HBV infection, inactive HBV carriers and patients with resolved HBV infection. Patients with no infection were those with negative HBsAg and negative anti-HBc. Chronic hepatitis B was defined as being HBsAg and anti-HBc positive, and at least one of HBeAg positive and HBV DNA positive. Those with positive HBsAg positive, and both HBeAg and HBV-DNA negative were labeled as inactive HBsAg carriers. Patients with negative HBsAg while positive with either anti-HBe or anti-HBc were subject to resolved HBV infection [24, 25].

#### Other parameters

Blood samples for measurement of serum CA199, liver function and serum amylase were obtained at the diagnosis. Medical records of smoking status and alcohol consumption of the patients were also reviewed.

#### Verification for clinical materials

All the cases were first reviewed by two doctors at each participating hospital, and then the clinical data was transferred to Shanghai Cancer Center. Two experienced doctors at Shanghai Cancer Center went through these clinical materials to determine the inclusion or the exclusion of the cases. If divergent views arise, a third doctor would be consulted for the final assessment of inclusion or exclusion.

### Outcome ascertainment

#### Synchronous liver metastasis

Up to now, there has been no established definition for synchronous liver metastasis from PDAC. In this study, we defined synchronous liver metastasis as follows:

Synchronous liver metastasis is defined as the detection of liver metastasis at the initial diagnosis of PDAC. The PDAC should be pathologically confirmed and the liver metastasis should be validated by imaging examination, including ultrasound, CT, MRI and PET-CT scan.

#### Overall survival and metachronous liver metastasis

All the patients in this study underwent the regular follow-up evaluations. Survival condition was actively followed up every month. For deceased patients, dates of death were obtained from their family members *via* telephone if the patients died after discharge or were recorded immediately if the patients died during hospitalization. The follow-up ended after the obtainment of death date.

Imaging evaluations were performed every 2 months within the first 3 years and every 3 months thereafter, of which chest X-ray, abdominal ultrasonography, triphasic cross-sectional abdominal CT or MRI were included. The images were initially reviewed by an experienced radiologist at each participating hospital and then re-evaluated by a radiologist at Shanghai Cancer Center. If inconsistent evaluation were reached by the first two doctors, a third radiology specialist at Shanghai Caner Center would be invited to give the conclusive evaluation.

Metachronous liver metastasis is defined as that liver metastasis can’t be found *via* imaging examination at the initial diagnosis of PDAC while can only be found during the follow-up imaging examinations, regardless of the time after the initial diagnosis. For example, liver metastasis detected one month or twelve months after the initial diagnosis of PDAC were both considered as metachronous liver metastasis.

### Statistical analysis

The statistical analyses were performed with SAS statistical software (version 9.4; SAS Institute). *P* values < 0.05 (two-sided probability) were interpreted as statistically significant.

Difference of synchronous liver metastasis rates between HBsAg positive and negative groups in stage IV patents were evaluated by chi-square test. A multivariate Logistic regression model was performed to analyze the association between HBV status and synchronous liver metastasis.

Crude metachronous liver metastasis rates of stage III patients were calculated based on the time interval from the date of diagnosis of pancreatic cancer to liver metastasis ascertained at follow-up stage, using the actuarial method that was usually performed in colorectal cancers [26]. Patients who died of undercurrent disease were censored at time of death, and patients who developed liver metastasis were censored at the time of occurrence. A multivariate analysis was performed using a Cox proportional hazards regression model to analyze the association between HBV status and metachronous liver metastasis.

Overall survival (OS) was the time interval from the date of diagnosis to death or to the last date of follow-up. OS curves were plotted with the Kaplan-Meier method, and differences between subgroups were evaluated by log-rank test. Cox regression models were used for univariate and multivariate analyses.

## RESULTS

### Cohort characteristics

A total of 1526 cases (833 from SCC, 486 from CHH, and 207 from RJH) that consisted of 553 females (36.2%) and 973 males (63.8%) were qualified for the analyses. Mean age at diagnosis was 58.6 years. Patients of stage III and IV were 595 (39.0%) and 931 (61.0%), respectively.

Non HBV infection was identified in 833 patients (54.6%). CHB was identified in 61 patients (4.0%). Inactive HBV carrier was identified in 83 patients (5.4%), and resolved HBV infection was identified in 549 patients (36.0%). Thus, 144 (9.4%) patients were enrolled in HBsAg positive group (CHB +IC), and 1382 (81.6%) patients were classified into HBsAg negative group (resolved HBV infection + non HBV infection). Detailed baseline characteristics are listed in Table 1. Proportions of CHB, IC, resolved HBV infection and non HBV infection are comparable between stage 3 and stage 4 patients, male and female patients, smoker and non-smoker patients, as well as alcohol and non-alcohol patients.

**Table 1:** Cohort Characteristics

	Total	SCC	CHH	RJH
**Age, n (%)**				
<60	815 (53.4)	443 (53.2)	252 (51.9)	120 (58.0)
≥60	711 (46.6)	390 (46.8)	234 (48.1)	87 (42.0)
**Sex, n (%)**				
Male	973 (63.8)	542 (65.1)	298 (61.3)	133 (64.3)
Female	553 (36.2)	291 (34.9)	188 (38.7)	74 (35.7)
**Smoking, n (%)**				
Yes	733 (48.4)	423 (51.1)	214 (44.4)	96 (46.6)
No	782 (51.6)	404 (48.9)	268 (55.6)	110 (53.4)
**Alcohol, n (%)**				
Yes	212 (14.0)	124 (14.9)	59 (12.3)	29 (14.1)
No	1305 (86.0)	707 (85.1)	422 (87.7)	176 (85.9)
**Stage, n (%)**				
Stage III	595 (39.0)	309 (37.1)	200 (41.2)	86 (41.5)
Stage IV	931 (61.0)	524 (62.9)	286 (58.8)	121 (58.5)
**HBV Status, n (%)**				
Chronic Hepatitis B	61 (4.0)	31 (3.7)	21 (4.3)	9 (4.3)
Inactive carrier	83 (5.4)	45 (5.4)	30 (6.2)	8 (3.9)
Non infection	833 (54.6)	486 (58.3)	242 (49.8)	105 (50.7)
Resolved infection	549 (36.0)	271 (32.5)	193 (39.7)	85 (41.1)
**ALT or AST, n (%)**				
Normal	1274 (83.5)	689 (82.7)	411 (84.6)	174 (84.1)
Elevated	252 (16.5)	144 (17.3)	75 (15.4)	33 (15.9)
**Liver cirrhosis, n (%)**				
Yes	10 (0.7)	5 (0.6)	3 (0.6)	2 (1.0)
No	1516 (99.3)	828 (99.4)	483 (99.4)	205 (99.0)
**ALB, n (%)**				
Normal	1226 (80.3)	672 (80.7)	385 (79.2)	169 (81.6)
Low	300 (19.7)	161 (19.3)	101 (20.8)	38 (18.4)
**CA199, n (%)**				
Normal	325 (21.3)	186 (22.3)	93 (19.1)	46 (22.2)
Elevated	1201 (78.7)	647 (77.7)	393 (80.9)	161 (77.8)

*SCC, Shanghai Cancer Center; CHH, Changhai Hospital; RJH, Ruijin Hospital

### Synchronous liver metastasis

Among stage IV patients, synchronous liver metastasis status were described at baseline. Synchronous liver metastasis was found in 810 (87%) patients. Synchronous liver metastasis rates in CHB, IC, resolved HBV infection and non HBV infection groups were 72.3%, 81.7%, 89.8% and 87.1%, respectively. Thus synchronous liver metastasis rates in HBsAg positive and negative groups were 77.6% and 88.3% (χ^2^ = 9.51, *P* = 0.0020), respectively.

In multivariable Logistic regression adjusted for other confounders, CHB was inversely associated with synchronous liver metastasis compared to non HBV infection (OR 0.41, 95% CI 0.19-0.85). IC and Resolved HBV infection showed no significant association with synchronous liver metastasis compared to non HBV infection. Detailed univariate and multivariate analyses are listed in Table 2.

**Table 2:** Factors Associated with the Risk of Synchronous Liver Metastasis for Stage IV Pancreatic Cancer (Univariate and Multivariate Logistic Regression Model)

HBV Status	Unadjusted		Adjusted*	
	OR	95% CI	OR	95% CI
Chronic Hepatitis B vs. Non infection	0.39	0.19-0.77	0.41	0.19-0.85
Inactive carrier vs. Non infection	0.66	0.33-1.33	0.61	0.29-1.26
Resolved infection vs. Non infection	1.30	0.84-2.03	1.31	0.83-2.07

* Adjusted for center (SCC, CHH, RJH), age at diagnosis (<60, ≥60), sex, current smoking status, alcohol intake, ALT or AST (normal, elevated), ALB (normal, low), and CA-199 (normal, elevated).

### Metachronous liver metastasis

Among 595 stage III patients, 186 (31.3%) developed a metachronous liver metastasis during follow up period after diagnosis. The overall actuarial cumulative rate was 6.2% at 3 months, 18.1% at 6 months, and 37.3% at 12 months. In different HBV status, 1 (7.1%) patent in CHB group, 4 (17.4%) in IC group, 64 (31.3%) in resolved HBV infection group and 117 (34.1%) in Non HBV infection group developed a metachronous liver metastasis. The 12-month cumulative rates were 0, 20.0%, 30.2% and 44.5% in the four HBV status subgroups, respectively. Association between HBV status and metachronous liver metastasis was analyzed in a multivariate Cox model to obtain a relative risk of liver metastasis adjusted for other covariables (Table 3). CHB (HR = 0.11, 95% CI 0.02-0.82) is considered as a protective factor of metachronous liver metastasis compared to Non HBV infection.

**Table 3:** Factors Associated with the Risk of Metachronous Liver Metastasis for Stage III Pancreatic Cancer (Univariate and Multivariate Cox Model)

HBV Status	Unadjusted			Adjusted*		
	HR	95% CI	*P*-value	HR	95% CI	*P*-value
Chronic Hepatitis B vs. Non infection	0.11	0.02-0.76	0.0255	0.11	0.02-0.82	0.0313
Inactive carrier vs. Non infection	0.46	0.17-1.23	0.1220	0.44	0.16-1.22	0.1139
Resolved infection vs. Non infection	0.82	0.60-1.11	0.1952	0.83	0.61-1.13	0.2378

* Adjusted for center (SCC, CHH, RJH), age at diagnosis (<60, ≥60), sex, current smoking status, alcohol intake, ALT or AST (normal, elevated), ALB (normal, low), and CA-199 (normal, elevated).

### Overall survival

The total follow-up period of this study lasted ten years from 2004 to 2014.The median follow-up for all patients was 6.2 months (range, 0.4 to 98.1 months). During this period, 1362 patients (89%) died, and 164 patients (11%) were censored. The median survival of stage III patients was 9.0 months, with 3-, 6- and 12-month survival rates of 94.5%, 73.0% and 28.5% respectively. The median survival of stage IV patients was 5.3 months, with 3-, 6- and 12-month survival rates of 79.3%, 42.6% and15.0% respectively.

**Figure 1:**
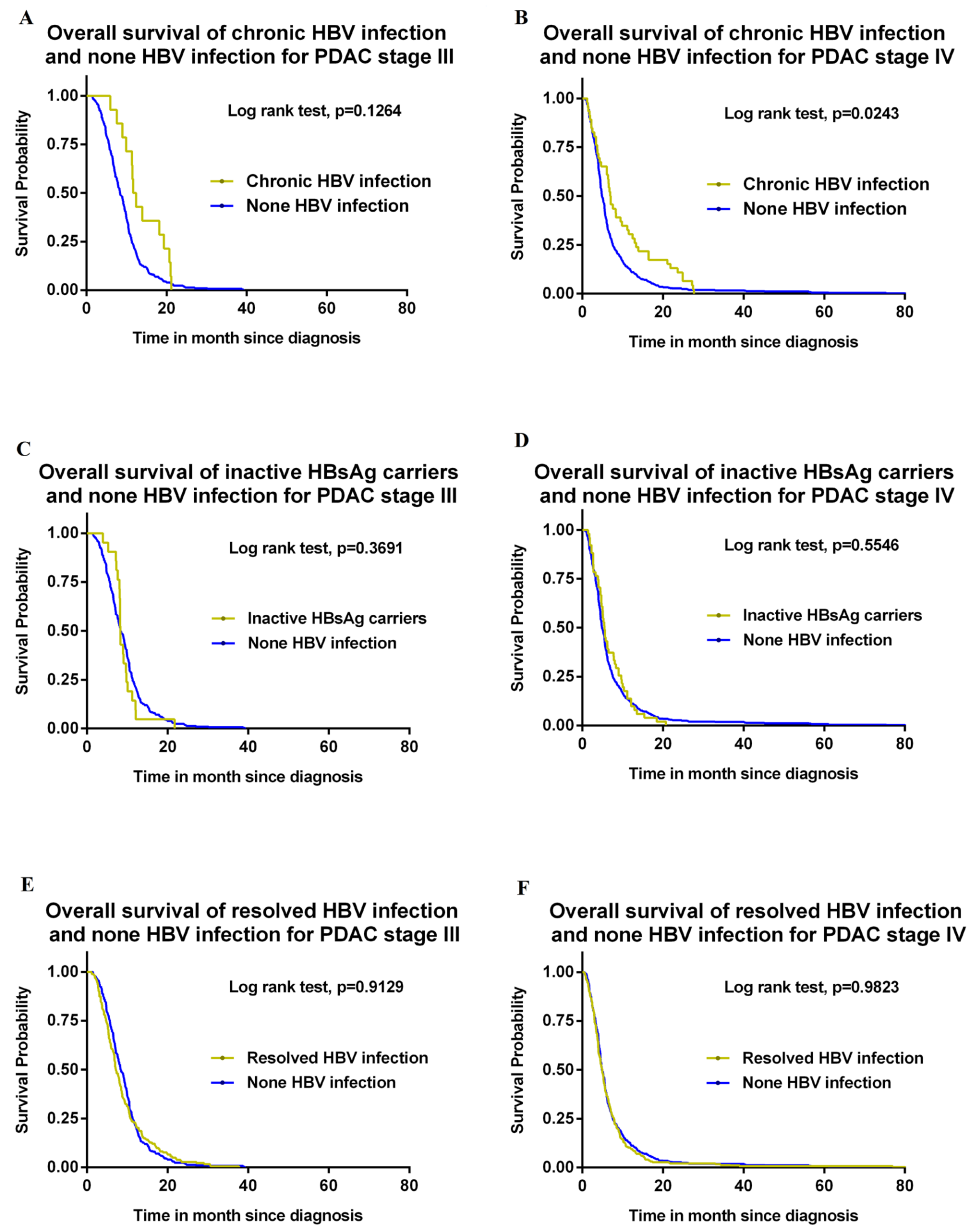
**A.** Overall survival of chronic HBV infection and none HBV infection for PDAC stage III. **B.** Overall survival of chronic HBV infection and none HBV infection for PDAC stage IV. **C.** Overall survival of inactive HBsAg carriers and none HBV infection for PDAC stage III. **D.** Overall survival of inactive HBsAg carriers and none HBV infection for PDAC stage IV. **E.** Overall survival of resolved HBV infection and none HBV infection for PDAC stage III. **F.** Overall survival of resolved HBV infection and none HBV infection for PDAC stage IV

The survival curves of CHB *vs*. non HBV infection, IC *vs*. non HBV infection and resolved HBV infection *vs*. non HBV infection plotted with Kaplan-Meier method were shown in Figure 1. The stage IV CHB patients had significantly longer overall survival than patients without HBV infection (*P* = 0.0243).

Association between HBV status and overall survival was analyzed in a multivariate Cox model adjusted for other covariables (Table 4). For stage IV patients, CHB (HR = 0.70, 95% CI 0.51-0.95) is considered as a protective factor of overall survival compared to Non HBV infection.

**Table 4:** Factors Associated with the Risk of Overall Survival for Stage III and Stage IV Pancreatic Cancer (Univariate and Multivariate Cox Model)

HBV Status	Unadjusted			Adjusted*		
	HR	95% CI	*P* -value	HR	95% CI	*P*-value
**Stage III (n=595)**						
Chronic Hepatitis B vs. Non infection	0.68	0.40-1.16	0.1575	0.64	0.37-1.10	0.1051
Inactive carrier vs. Non infection	1.19	0.76-1.85	0.4488	1.19	0.76-1.87	0.4505
Resolved infection vs. Non infection	0.99	0.82-1.19	0.9016	0.97	0.81-1.18	0.7814
**Stage IV (n=931)**						
Chronic Hepatitis B vs. Non infection	0.71	0.52-0.96	0.0246	0.70	0.51-0.95	0.0227
Inactive carrier vs. Non infection	0.92	0.69-1.23	0.5865	0.88	0.65-1.18	0.3905
Resolved infection vs. Non infection	1.00	0.86-1.16	0.9820	0.99	0.85-1.15	0.9078
**Pooled Stage III and Stage IV (n=1526)**						
Chronic Hepatitis B vs. Non infection	0.75	0.58-0.98	0.0355	0.74	0.57-0.97	0.0283
Inactive carrier vs. Non infection	1.07	0.841.36	0.5941	1.04	0.82-1.33	0.7447
Resolved infection vs. Non infection	1.01	0.90-1.14	0.8425	1.00	0.89-1.12	0.9467

* Adjusted for center (SCC, CHH, RJH), age at diagnosis (<60, ≥60), sex, current smoking status, alcohol intake, ALT or AST (normal, elevated), ALB (normal, low), and CA-199 (normal, elevated).

## DISCUSSION

 Our study showed that patients with CHB had significant lower rate of liver metastasis compared with the non-HBV infection group, which indicated that HBV infection with active replication might serve as a protective factor for liver metastasis in PDAC. Additionally, the overall survival of patients in the stage IV CHB group is significantly longer than that in the non-HBV infection group, indicating the essential role of CHB in the outcome among PDAC patients and its prognostic indicator for overall survival among PDAC patients.

Some studies have demonstrated that the incidence of liver metastasis in colorectal cancer was lower in patients with HBV infection compared to the uninfected ones [20-23]. Our study showed a similar trend in synchronous and metachronous liver metastasis in the unresectable PDAC. In the present study, CHB is associated with less metachronous liver metastasis in PDAC. To some extent, it even serve as a protective factor, extending the overall survival of these patients. However, Wei et al. [27] found that HBV infection increased synchronous liver metastasis incidence. Nevertheless, our study gave out a different answer to the synchronous liver metastasis regarding HBV status.

 Moreover, prevalence of metachronous liver metastases showed a similar tendency as that in synchronous metastases. The association between HBV and liver metastasis in our study is consistent with some previous reports in colorectal cancer while inconsistent with Wei's study. Notably, Wei's study enrolled both the operable and the non-operable subjects. Patients with HBV infection may receive surveillance ultrasound every 3-6 months as a means for detection of HCC at early stage. Since routine ultrasound performance can help detect PDAC at early stage, it is sensible that PDAC patients with HBV infection have better prognosis by means of operation due to early detection. Therefore, the underlying possibility can be a misleading factor affecting the outcome of OS in the patients at different cancer stages in the study led by Wei [27]. It has to be noted that the prognosis of patients undergoing surgery are more likely to be influenced by parameters like surgical margin, lymphatic metastasis and nerve invasion, as reported by numerous studies. Therefore, the current study only enrolled non-operable patients to exclude the unknown influences. To the best of our knowledge, our cohort study is the largest one centering on the effect of HBV infection in PDAC patients up to date.

It has been demonstrated that the existence of HBV virus in pancreatic juice, bile and tissue in patients with HBV positivity, which suggested HBV may be one of the major etiological factors for PDAC [14, 16]. Some studies suggested that the past exposure to HBV infection(resolved HBV infection) may enhance the risk of suffering from PDAC [17], while some other studies suggested that HBV infection may not be a risk factor for the pathogenesis of PDAC [18, 19, 28]. Currently, whether HBV plays a prognostic role in the progression of advanced PDAC remains controversial and needs to be further explored. It has to be noted that this cohort study focused on the impact of current and previous exposure to HBV. Our study found that rather than a detrimental factor accelerating liver metastasis, HBV infection may act as a protective factor to hinder liver metastasis in patients with advanced PDAC. And these findings may contradict with HBV's carcinogenic role. Prior to this study, we conjectured that patients with current or past exposure to HBV infection maybe have poorer OS or higher incidence of liver metastasis as compared to their non-infected counterparts. However, our study may suggest that HBV may be a favorable prognostic factor for unresectable PDAC during its progression.

In addition, similar observation was also found in colorectal cancer. Song et al [22] found that the incidence of liver metastasis in the HBV infection group was significantly lower than that in the non-infection group. Therefore, we speculated that HBV may serve as a beneficial factor in PDAC patients, possibly by undermining the invasive and metastatic ability of the primary tumor. However, the underlying mechanisms remain to be explored.

As is generally recognized, long-term persistence of HBV infection can cause an inflammatory microenvironment in the liver [29], which may trigger enhanced immune defense [30, 31]. The boosted immune responses were assumed to be beneficial in the inhibition of hepatic metastasis among PDAC patients [30, 31]. It has been reported that HBV replication can enhance the cytotoxic ability of a series of immune cells, the major players involving in the immune responses [32, 33]. These functional immune cells are featured by Kupffer cells, NK cells, NKT cells, dendritic cells and CD8+CD122+ cells [32, 33]. Several *in vivo* studies have found that NK cells, an important type of immune cells that reside in the liver sinusoids, can adhere to sinusoidal endothelial cells and Kupffer cells, therefore exerting its antimetastatic effect [34, 35]. Metastases of hematogenous tumor to the liver can also be inhibited by NKT cells stimulated with recombinant interleukin-12 (IL-12) [36]. Meanwhile, tumor growth in the liver can be inhibited by *α*-GalCer, inductor of bystander CD8+ CD122+ T cells and tumor-specific cytotoxic CD8+ T cells [36, 37]. These cells altogether formed a niche in the liver of patients with CHB, rendering the liver not a proper site for metastasis.

In addition, HBV replication may boost tumor necrosis factor a (TNF-a) secretion *via* regulating hepatocytes and immune cells residing in the liver [38]. In view that HBV can also replicate in pancreas [14, 16], we speculate that HBV also plays a role in the biological behavior of PDAC, thereby attenuating tumor invasiveness. Pitifully, there was no data reflecting the status of HBV infection in the pancreas or its immunity variation in this study. Therefore, further investigations are needed to explore the immunity microenvironment in pancreatic cancerous tissue in PDAC patients with CHB.

Despite increased survival in PDAC patients with the treatment of nab-Paclitaxel plus Gemcitabine [39], the prognosis of advanced PDAC remains grim. Up to now, there have been no studies demonstrating the benefit of anti-viral medication in patients with CHB infection. Contrary to the conventional view, we assumed that CHB may be a protective factor for unresectable PDAC patients. Therefore, the anti-viral regimen may not be necessary for PDAC patients with CHB as it may even worsen the prognosis. Further rigorously designed clinical trials are needed to confirm our conjecture, which may provide new insights into the prevention of liver metastasis and pr/ioritize the treatment strategy to extend PDAC patients’ life expectancies.

## LIMITATION OF THIS STUDY

It has been demonstrated that HBV exists in both cancerous tissue and noncancerous pancreatic tissue in PDAC patients. In this cohort, evidence suggests a link between HBV serum markers and the progression of advanced PDAC, but data directly reflecting the status of HBV infection in the pancreas or its immunity variation lacked. Further investigations are needed to explore the immunity microenvironment in pancreatic cancerous tissue in PDAC patients with CHB. This may help more directly understand the invasiveness of PDAC influenced by HBV infection. Meanwhile, CHB is not significantly associated with OS for stage III patients, which may be due to lacking of power. Thus, more participating centers and more subjects may be needed to confirm the protective role of CHB for stage III PDAC patients.

## CONCLUSIONS

The present study demonstrated that CHB infection may decrease the risks of synchronous or metachronous liver metastasis, and prolong overall survival in advanced PDAC patients. However, more researches are warranted to investigate the underlying mechanisms involved. And whether anti-HBV medication could benefit advanced PDAC patients needs to be proven in future clinical trials.

## References

[1] Siegel R, Naishadham D, Jemal A (2013). Cancer statistics, 2013. CA: A Cancer Journal for Clinicians.

[2] Chen W, Zheng R, Zhang S, Zhao P, Zeng H, Zou X (2014). Report of cancer incidence and mortality in China, 2010. Ann Transl Med.

[3] Li D, Xie K, Wolff R, Abbruzzese JL (2004). Pancreatic cancer. The Lancet.

[4] Hartwig W, Werner J, Jager D, Debus J, Buchler MW (2013). Improvement of surgical results for pancreatic cancer. The Lancet Oncology.

[5] Chao YJ, Sy ED, Hsu HP, Shan YS (2014). Predictors for resectability and survival in locally advanced pancreatic cancer after gemcitabine-based neoadjuvant therapy. BMC Surg.

[6] Zhou G, Niu L, Chiu D, He L, Xu K (2012). Changes in the expression of serum markers CA242, CA199, CA125, CEA, TNF-alpha and TSGF after cryosurgery in pancreatic cancer patients. Biotechnol Lett.

[7] Thio CL, Guo N, Xie C, Nelson KE, Ehrhardt S (2015). Global elimination of mother-to-child transmission of hepatitis B: revisiting the current strategy. The Lancet Infectious Diseases.

[8] Cui Y, Jia J (2013). Update on epidemiology of hepatitis B and C in China. J Gastroen Hepatol.

[9] Yang HI, Yuen MF, Chan HL, Han KH, Chen PJ, Kim DY, Ahn SH, Chen CJ, Wong VW, Seto WK (2011). Risk estimation for hepatocellular carcinoma in chronic hepatitis B (REACH-B): development and validation of a predictive score. The Lancet Oncology.

[10] Yi HZ, Chen JJ, Cen H, Yan W, Tan XH (2014). Association between infection of hepatitis B virus and onset risk of B-cell non-Hodgkin's lymphoma: a systematic review and a meta-analysis. Med Oncol.

[11] Ulcickas YM, Quesenberry CJ, Guo D, Caldwell C, Wells K, Shan J, Sanders L, Skovron ML, Iloeje U, Manos MM (2007). Incidence of non-Hodgkin's lymphoma among individuals with chronic hepatitis B virus infection. Hepatology.

[12] Brown KE, Tisdale J, Barrett AJ, Dunbar CE, Young NS (1997). Hepatitis-associated aplastic anemia. New Engl J Med.

[13] Lai KN, Li PK, Lui SF, Au TC, Tam JS, Tong KL, Lai FM (1991). Membranous nephropathy related to hepatitis B virus in adults. New Engl J Med.

[14] Hoefs JC, Renner IG, Askhcavai M, Redeker AG (1980). Hepatitis B surface antigen in pancreatic and biliary secretions. Gastroenterology.

[15] Jin Y, Gao H, Chen H, Wang J, Chen M, Li G, Wang L, Gu J, Tu H (2013). Identification and impact of hepatitis B virus DNA and antigens in pancreatic cancer tissues and adjacent non-cancerous tissues. Cancer Lett.

[16] Shimoda T, Shikata T, Karasawa T, Tsukagoshi S, Yoshimura M, Sakurai I (1981). Light microscopic localization of hepatitis B virus antigens in the human pancreas. Possibility of multiplication of hepatitis B virus in the human pancreas. Gastroenterology.

[17] Hassan MM, Li D, El-Deeb AS, Wolff RA, Bondy ML, Davila M, Abbruzzese JL (2008). Association between hepatitis B virus and pancreatic cancer. J Clin Oncol.

[18] de Gonzalez AB, Jee SH, Engels EA (2009). No association between hepatitis B and pancreatic cancer in a prospective study in Korea. J Clin Oncol.

[19] Chang MC, Chen CH, Liang JD, Tien YW, Hsu C, Wong JM, Chang YT (2014). Hepatitis B and C viruses are not risks for pancreatic adenocarcinoma. World J Gastroenterol.

[20] Qian HG, Hao CY (2014). Hepatitis B virus infection is an independent factor influencing the occurrence of liver metastasis in colorectal cancer: a retrospective analysis of 1413 cases. Hepatogastroenterology.

[21] Qiu HB, Zhang LY, Zeng ZL, Wang ZQ, Luo HY, Keshari RP, Zhou ZW, Xu RH (2011). HBV infection decreases risk of liver metastasis in patients with colorectal cancer: A cohort study. World J Gastroenterol.

[22] Song E, Chen J, Ou Q, Su F (2001). Rare occurrence of metastatic colorectal cancers in livers with replicative hepatitis B infection. Am J Surg.

[23] Wang FS, Shao ZG, Zhang JL, Liu YF (2012). Colorectal liver metastases rarely occur in patients with chronic hepatitis virus infection. Hepatogastroenterology.

[24] Liang TJ (2009). Hepatitis B: the virus and disease. Hepatology.

[25] Lok AS, McMahon BJ (2007). Chronic hepatitis B. Hepatology.

[26] Manfredi S, Lepage C, Hatem C, Coatmeur O, Faivre J, Bouvier AM (2006). Epidemiology and management of liver metastases from colorectal cancer. Ann Surg.

[27] Wei XL, Qiu MZ, Chen WW, Jin Y, Ren C, Wang F, Luo HY, Wang ZQ, Zhang DS, Wang FH, Li YH, Xu RH (2013). The status of HBV infection influences metastatic pattern and survival in Chinese patients with pancreatic cancer. J Transl Med.

[28] Tang J, Sharma R, Lamerato L, Sheehan M, Krajenta R, Gordon SC (2014). Is previous exposure to hepatitis B a risk factor for pancreatic cancer or hepatocellular carcinoma?. J Clin Gastroenterol.

[29] Kennedy PT, Sandalova E, Jo J, Gill U, Ushiro-Lumb I, Tan AT, Naik S, Foster GR, Bertoletti A (2012). Preserved T-cell function in children and young adults with immune-tolerant chronic hepatitis B. Gastroenterology.

[30] Giersch K, Allweiss L, Volz T, Helbig M, Bierwolf J, Lohse AW, Pollok JM, Petersen J, Dandri M, Lutgehetmann M (2015). Hepatitis Delta co-infection in humanized mice leads to pronounced induction of innate immune responses in comparison to HBV mono-infection. J Hepatol.

[31] Ferrari C (2015). HBV and the immune response. Liver Int.

[32] Motegi A, Kinoshita M, Inatsu A, Habu Y, Saitoh D, Seki S (2008). IL-15-induced CD8+CD122+ T cells increase antibacterial and anti-tumor immune responses: implications for immune function in aged mice. J Leukoc Biol.

[33] Diehl L, Schurich A, Grochtmann R, Hegenbarth S, Chen L, Knolle PA (2008). Tolerogenic maturation of liver sinusoidal endothelial cells promotes B7-homolog 1-dependent CD8+ T cell tolerance. Hepatology.

[34] Rushfeldt C, Sveinbjornsson B, Seljelid R, Smedsrod B (1999). Early events of hepatic metastasis formation in mice: role of Kupffer and NK-cells in natural and interferon-gamma-stimulated defense. J Surg Res.

[35] Takahashi M, Ogasawara K, Takeda K, Hashimoto W, Sakihara H, Kumagai K, Anzai R, Satoh M, Seki S (1996). LPS induces NK1. 1+ alpha beta T cells with potent cytotoxicity in the liver of mice via production of IL-12 from Kupffer cells. J Immunol.

[36] Li M, Sun R, Xu L, Yin W, Chen Y, Zheng X, Lian Z, Wei H, Tian Z (2015). Kupffer Cells Support Hepatitis B Virus-Mediated CD8+ T Cell Exhaustion via Hepatitis B Core Antigen-TLR2 Interactions in Mice. J Immunol.

[37] Dolina JS, Braciale TJ, Hahn YS (2014). Liver-primed CD8+ T cells suppress antiviral adaptive immunity through galectin-9-independent T-cell immunoglobulin and mucin 3 engagement of high-mobility group box 1 in mice. Hepatology.

[38] Chyuan IT, Tsai HF, Tzeng HT, Sung CC, Wu CS, Chen PJ, Hsu PN (2015). Tumor necrosis factor-alpha blockage therapy impairs hepatitis B viral clearance and enhances T-cell exhaustion in a mouse model. Cellular and Molecular Immunology.

[39] Von Hoff DD, Ervin T, Arena FP, Chiorean EG, Infante J, Moore M, Seay T, Tjulandin SA, Ma WW, Saleh MN, Harris M, Reni M, Dowden S (2013). Increased survival in pancreatic cancer with nab-paclitaxel plus gemcitabine. N Engl J Med.

